# Supplementary material for: Male-Biased Adult Production of the Striped Fruit Fly, Zeugodacus scutellata, by Feeding dsRNA Specific to Transformer-2
Source: Insects. 2020 Mar 28;11(4):211. doi: 10.3390/insects11040211 (PMC7240746; doi:10.3390/insects11040211)
Supplement: Supplementary file 1 [file insects-11-00211-s001.zip › insects-749425-figure S1.pdf]

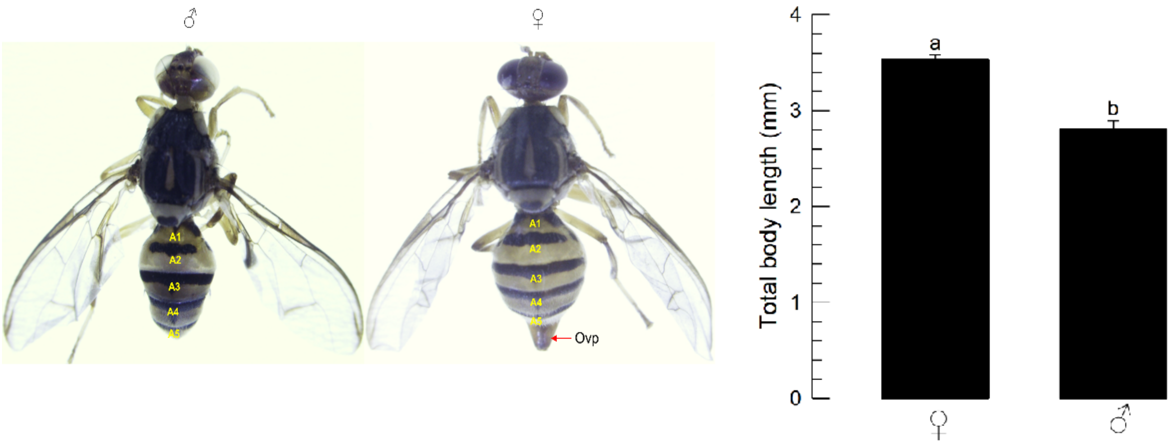

(**A**)

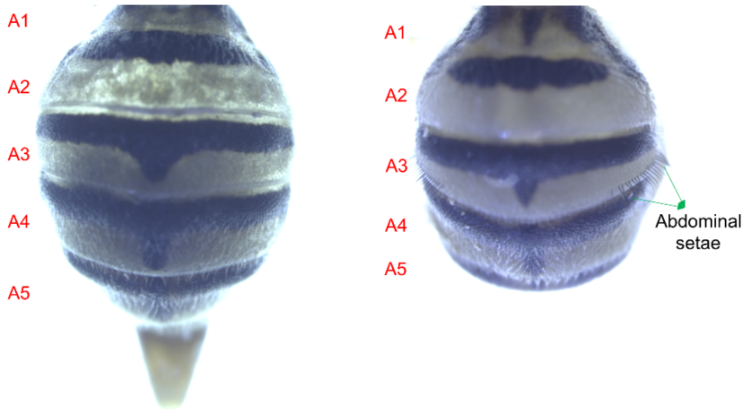

(**B**)

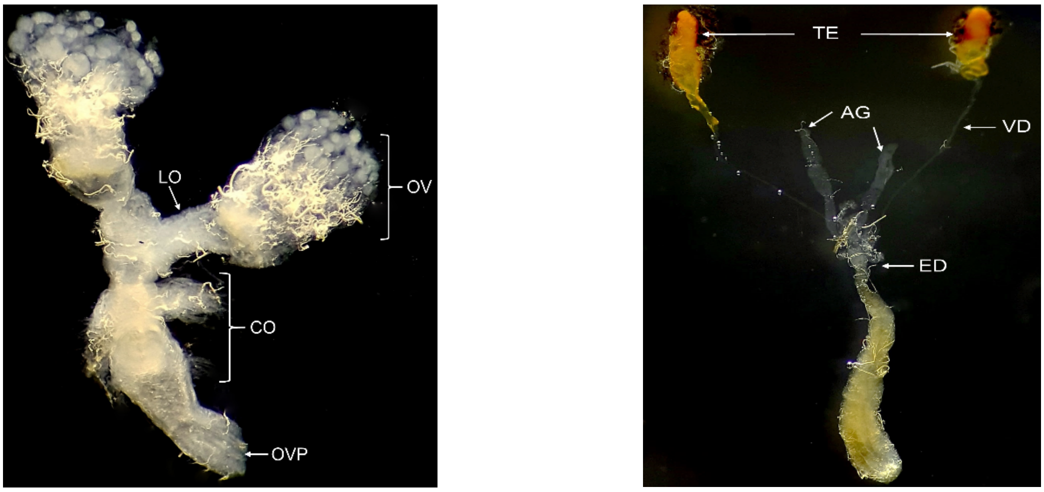

(**C**)

Figure S1. Sexual morphs of *Z. scutellata* adults. (**A**) Comparison of total body length from head to abdomen between males and females. Ovipositor (‘OVP’) was not included in the measurement of body length in females. Ten individuals of each sex were used. Different letters above standard deviation bars indicate significant difference among means at Type I error = 0.05 (LSD test). (**B**) Abdominal pleural setae on males. Abdominal segments are labeled with A1-A5 in both sexes. (**C**) Internal reproductive organs. Ovary (OV) and testis (TE) structures were obtained from 20 days old female and male adults. A pair of ovaries are linked from common oviduct (‘CO’) via lateral oviduct

(‘LO’). Two testes are linked to ejaculatory duct (‘ED’) through vas deferens (‘VD’) and ended with aedegus (‘AED’).
