# Supplementary material for: Male-Biased Adult Production of the Striped Fruit Fly, Zeugodacus scutellata, by Feeding dsRNA Specific to Transformer-2
Source: Insects. 2020 Mar 28;11(4):211. doi: 10.3390/insects11040211 (PMC7240746; doi:10.3390/insects11040211)
Supplement: Supplementary file 1 [file insects-11-00211-s001.zip › insects-749425-Table S1.pdf]

Table S1. Primers used in this study for RT-qPCR

Gene	Primer sequence (5' - 3')	Annealing temperature (°C)	Purposes
Tra2	GACATAGCGGATGCTAAA	52	RT-PCR RT-qPCR
	TTGCGACTGTGATAAGG		
	TAATACGACTCACTATAGGGAGAGACATAGCGGATGCTAAA	52	RNAi
	TAATACGACTCACTATAGGGAGATTGCGACTGTGATAAGG		
Actin	CAACACACCCGCCATGTATG	50	RT-PCR RT-qPCR
	ACGAGTAGCCACGTTCAGTC		
